# The suppression of Antarctic bottom water formation by melting ice shelves in Prydz Bay

**DOI:** 10.1038/ncomms12577

**Published:** 2016-08-23

**Authors:** G. D. Williams, L. Herraiz-Borreguero, F. Roquet, T. Tamura, K. I. Ohshima, Y. Fukamachi, A. D. Fraser, L. Gao, H. Chen, C. R. McMahon, R. Harcourt, M. Hindell

**Affiliations:** 1 Institute for Marine and Antarctic Studies, University of Tasmania, Private Bag 129, Hobart, Tasmania 7001, Australia; 2 Antarctic Climate & Ecosystems Cooperative Research Centre, University of Tasmania, Private Bag 80, Hobart 7001, Australia; 3 Centre for Ice and Climate, Niels Bohr Institute, University of Copenhagen, Julianne Marie vej 30, Copenhagen 2100, Denmark; 4 Department of Meteorology, Stockholm University, Stockholm 106 91, Sweden; 5 National Institute of Polar Research, Tokyo 190-8518, Japan; 6 Sokendai (The Graduate University for Advanced Studies), Tokyo 190-8518, Japan; 7 Institute of Low Temperature Science, Hokkaido University Kita-19, Nishi-8, Sapporo 060-0819, Japan; 8 The First Institute of Oceanography, State Oceanic Administration, No. 6 Xianxialing Road, Qingdao 266061, China; 9 Sydney Institute of Marine Science, 19 Chowder Bay Road, Mosman, New South Wales 2088, Australia; 10 Department of Biological Sciences, Macquarie University, New South Wales 2109, Australia

## Abstract

A fourth production region for the globally important Antarctic bottom water has been attributed to dense shelf water formation in the Cape Darnley Polynya, adjoining Prydz Bay in East Antarctica. Here we show new observations from CTD-instrumented elephant seals in 2011–2013 that provide the first complete assessment of dense shelf water formation in Prydz Bay. After a complex evolution involving opposing contributions from three polynyas (positive) and two ice shelves (negative), dense shelf water (salinity 34.65–34.7) is exported through Prydz Channel. This provides a distinct, relatively fresh contribution to Cape Darnley bottom water. Elsewhere, dense water formation is hindered by the freshwater input from the Amery and West Ice Shelves into the Prydz Bay Gyre. This study highlights the susceptibility of Antarctic bottom water to increased freshwater input from the enhanced melting of ice shelves, and ultimately the potential collapse of Antarctic bottom water formation in a warming climate.

## Abstract (web summary)

Antarctic bottom water (AABW) production is critical to the global ocean overturning circulation. Here, the authors show new observations of AABW formation from seal CTD data in Prydz Bay, East Antarctica that highlights its susceptibility to increased freshwater input from the melting of ice shelves.

## Graphical abstract

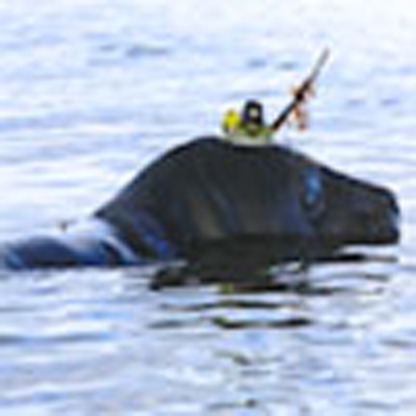

Antarctic bottom water (AABW) production is vital to the Earth's climate system and biogeochemical cycles^1^,^2^,^3^. AABW results from the downslope transport of cold dense shelf water (DSW) mixing with ambient water masses on the continental slope. The key mechanism for DSW formation is the enhanced sea ice production and subsequent salt-rejection in coastal polynyas^4^. These polynyas are found in discrete locations on the continental shelf around Antarctica, but only a few regions, for example, the Weddell Sea^5^, the Ross Sea^6^ and the Adélie and George V Land coast^7^,^8^,^9^, can export DSW with sufficient negative buoyancy to produce AABW. Recently, a fourth region of AABW production was confirmed immediately downstream from Prydz Bay (69°–81°E), after newly ventilated AABW was observed in canyons on the continental slope north of Cape Darnley^10^, in the eastern sector of the Weddell–Enderby Basin (Fig. 1). This Cape Darnley bottom water (CDBW) is an important ventilator of the abyssal ocean, estimated to contribute 13–30% of Atlantic AABW production^10^.

Decades of speculation about a DSW source in Prydz Bay^11^,^12^,^13^ ended after satellite-derived estimates of enhanced sea ice production (∼180 km^3^ per year)^14^,^15^ in the Cape Darnley polynya (CDP) were directly linked to very saline DSW (>34.8) using data collected by instrumented southern elephant seals^16^ (*Mirounga leonina*). This led to the conclusion that the primary DSW source of CDBW was the Cape Darnley polynya^10^. There was evidence of a lower-salinity DSW source, attributed to the upstream Prydz Bay region, although the available seal data in Prydz Bay from June 2011 showed a bottom salinity of less than 34.6, significantly less than DSW (34.8–34.9) from Cape Darnley. However, there is an apparent contradiction between the supposedly minor contribution of DSW from Prydz Bay and the fact that Prydz Bay has three medium-sized polynya regions (Fig. 1, Supplementary Fig. 1) collectively representing 30% more sea ice production than Cape Darnley (Supplementary Table 1). It also left open the question of whether Prydz Bay polynyas had any role in the pre-conditioning and production of CDBW.

Here we focus on the role of the Prydz Bay polynyas and ice shelves in the formation of DSW to clarify Prydz Bay's contribution to CDBW. We use a further 2 years of salinity and temperature observations from instrumented seals in 2012–2013 that expand both the spatial and seasonal coverage of DSW in Prydz Bay. Extended periods of concentrated profiling of the water column around the periphery of the Prydz Bay Gyre (PBG) are now available. These unique observations in the key polynyas of Barrier Bay, Davis and MacKenzie Bay, and across the outflow region through Prydz Channel upstream of Cape Darnley, allows us to present a comprehensive picture of the evolution of DSW formation and export from Prydz Bay. Documenting the impact of ice shelves on polynya-driven AABW production, this study is timely given the growing evidence of widespread thinning of both West and East Antarctic coastal ice sheet margins^17^,^18^,^19^,^20^.

## Results

### The PBG and distribution of shelf water masses

The ocean circulation in Prydz Bay consists of a large cyclonic gyre, centred in a deep channel, known as the Amery Depression^12^,^21^. The PBG is associated with a relatively narrow coastal current that runs along the Amery Ice Shelf calving front, and continues westward after leaving Prydz Bay. We use the geopotential height anomaly from the seal CTD data (April through May, 2011–12, Supplementary Fig. 2) to show the most data-rich representation of the PBG to-date (Fig. 2a). The geopotential height anomaly is calculated at 50 m referenced to 300 m (chosen to maximize the number of profiles available). Our inferred circulation agrees well with what was known about PBG, but suggests a more complex circulation along the ice shelf front than previously depicted. Recent observations^22^,^23^ and modelling studies^24^,^25^ suggest a similar cyclonic circulation in the Amery Ice Shelf ocean cavity (that is, inflow of shelf waters in the east and outflow of ice shelf water in the west). The robust nature of the ocean circulation is readily visible in the spatial distribution of the main shelf water masses that occupy Prydz Bay, namely modified circumpolar deep water (mCDW), ice shelf water (ISW) and DSW.

In the eastern region of Prydz Bay, mCDW (θ>−1.85 °C, 28.00<γ^n^<28.27) is the dominant water mass from April through May, intruding at intermediate depths and moving southeastward over the Four Ladies Bank towards Davis Station and the eastern Amery Ice Shelf front^15^ (Fig. 2b). Offshore on the continental slope, mCDW has a maximum temperature close to 0 °C and salinity higher than 34.65 (not shown). In contrast, only heavily modified mCDW is present over the continental shelf. The depth of the mCDW core is between 250 and 450 dbar (not shown) between ∼67°S and 68.5° S, descending up to 500–600 dbar at the ice shelf calving front^15^. While there is a distinct lack of mCDW in Barrier Bay, in the northeast corner of Prydz Bay, there is a small signal of re-circulated mCDW in the western half of Prydz Bay (Fig. 2b).

In the southwest region of Prydz Bay and along the western flank, ISW is the dominant water mass at intermediate depths from April through May (Fig. 2c). Typically, ISW temperatures are below the surface freezing point, forming a mixture of glacial meltwater from ocean/ice shelf interactions with ambient shelf waters. Here we define ISW with θ<−1.95 °C, which is sufficiently beneath the surface freezing point (∼−1.92 °C) to account for the accuracy of the temperature data. Previous summertime observations using a warmer definition of ISW (−1.90 °C) show a greater presence of ISW along the western flank of Prydz Bay^26^. We find that from April through October, the ISW signal in MacKenzie Bay from the seal data becomes diminished. This is likely due to a combination of the polynya-driven convection eroding the temperature signal of the ISW, and the seal data locations becoming concentrated in the northwest region, seemingly removed from the main ISW core to the east (Supplementary Fig. 3).

Beneath the Amery Ice shelf, mCDW and DSW forms two distinct ISW types but only the ISW formed from DSW (ISW_DSW_) has been observed outflowing through the western flank of the calving front^23^. Intense sampling of the seal data in the MacKenzie Bay polynya along the western half of the Amery Ice Shelf front detects this ISW_DSW_ at depths between 50 and 500 m (Fig. 2c). This cold signature can be found recirculating in Prydz Bay at deeper depths in the eastern Prydz Bay and out along the western flank through Prydz Channel, again following the broad circulation pattern of the PBG. ISW is fresher than its source water through the input of glacial meltwater and its mixing pathways on leaving the Amery Ice Shelf front has important consequences for polynya-driven DSW formation.

### Prydz Bay DSW formation and export through Prydz Channel

We examine the presence of DSW (neutral density *γ*^n^>28.27, practical salinity *S*>34.5) in the polynyas around Prydz Bay following the PBG circulation, from the northeast at the Barrier Bay polynya, followed by the Davis, MacKenzie Bay and Cape Darnley polynyas (Fig. 3a). The distribution of bottom-of-dive DSW shows an increase in the salinity of the DSW as it flows from the Barrier Bay/Davis polynyas (*S*<34.55), to the MacKenzie Bay polynyas (*S*<34.7). The latter is also observed along the western flank of Prydz Bay through Prydz Channel. As previously reported^10^, we find the most saline DSW in the Cape Darnley region (*S*>34.8).

Continuous diving by the seals within the key polynya regions over the autumn–winter period resembles profiling moorings and provides high temporal resolution observations of stratification and water mass properties. The seasonal time series of mean salinity at 300 m is shown in Fig. 3b. Barrier Bay shows a clear increase in salinity from <34.4 towards a peak of ∼34.55 in September (Fig. 3b, dark green line). The spatial sampling for the Davis polynya region was broader (not shown) and appears to start with more saline properties (Fig. 3b, yellow line) relative to Barrier Bay in May. However, there are also sharp incursions to fresher values that more closely resemble the DSW leaving Barrier Bay. While the deepest and most saline DSW within Barrier Bay is blocked from travelling south by the Four Ladies Bank, DSW<300 m is likely to mix across and flow towards the Amery Ice Shelf cavity.

**Figure 1:**
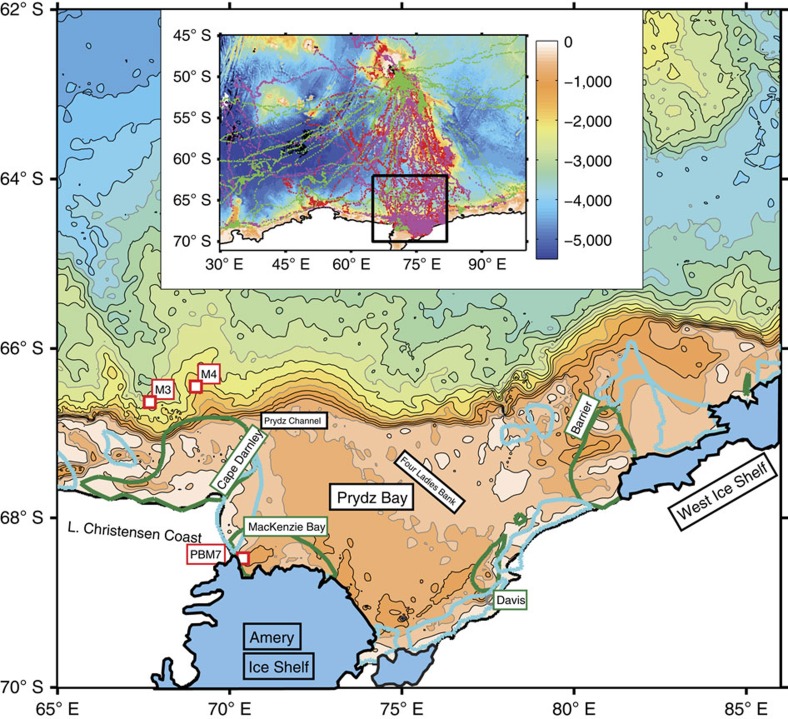
Study area of Prydz Bay and surrounds. Bathymetry from ETOP01 (http://www.ngdc.noaa.gov/mgg/global/global.html) with 250 m contour intervals. Major geographical features shown include Prydz Bay, Prydz Channel and the Four Ladies Bank, with the Amery and West Ice Shelves. Coastal polynyas are also shown using satellite-derived sea ice production estimates (dark green contours show 5 ma^−1^) from ERA-Interim data (1992–2014)^14^,^15^: Cape Darnley, MacKenzie Bay, Davis and Barrier Bay. Mean fast ice contours^37^ are shown as thick light blue contours. Instrumented mooring locations M3, M4 (ref. 10) and PBM7 are shown with red squares. Inset: large-scale bathymetry of Southern Indian Ocean (45–72° S) across the Antarctic margin (30–100° E) with elephant seal data locations from Davis Station deployments (red and magenta points for 2011 and 2012, respectively) and an Iles Kerguelen deployment (green points from 2013). Black box indicates the location of the study area shown.

The sampling of MacKenzie Bay polynya was predominantly north of the previous AMISOR mooring PBM7 (Fig. 1; light green dashed in Fig. 3b). Seals occupied the western-most part of this polynya in all 3 years (Supplementary Fig. 3b), and delivered an overlapping time series of salinity from mid-July to mid-September in 2012–2013 (Fig. 3b, blue lines). These time series show a sharp increase of 0.1–0.15 in mean salinity relative to the eastern polynya regions and the 2001–2002 PBM7 mooring, reaching a maximum >34.65 in September to October. These values match those from Fig. 3a traced north along the western flank of Prydz Bay and out through Prydz Channel (Fig. 3b, black squares). Finally, we show a time series from the Cape Darnley polynya, over a shorter time period (Fig. 3b, red line, May through June), where the salinity values track alongside those from MacKenzie Bay, but are offset by an increase in salinity of approximately 0.1–0.125. Additional sampling from the Cape Darnley polynya region (red dots) shows these DSW values continue on their trajectory past 34.8 by early July, in accordance with the values first reported in the confirmation of the primary source of Cape Darnley bottom water^10^.

**Figure 2:**
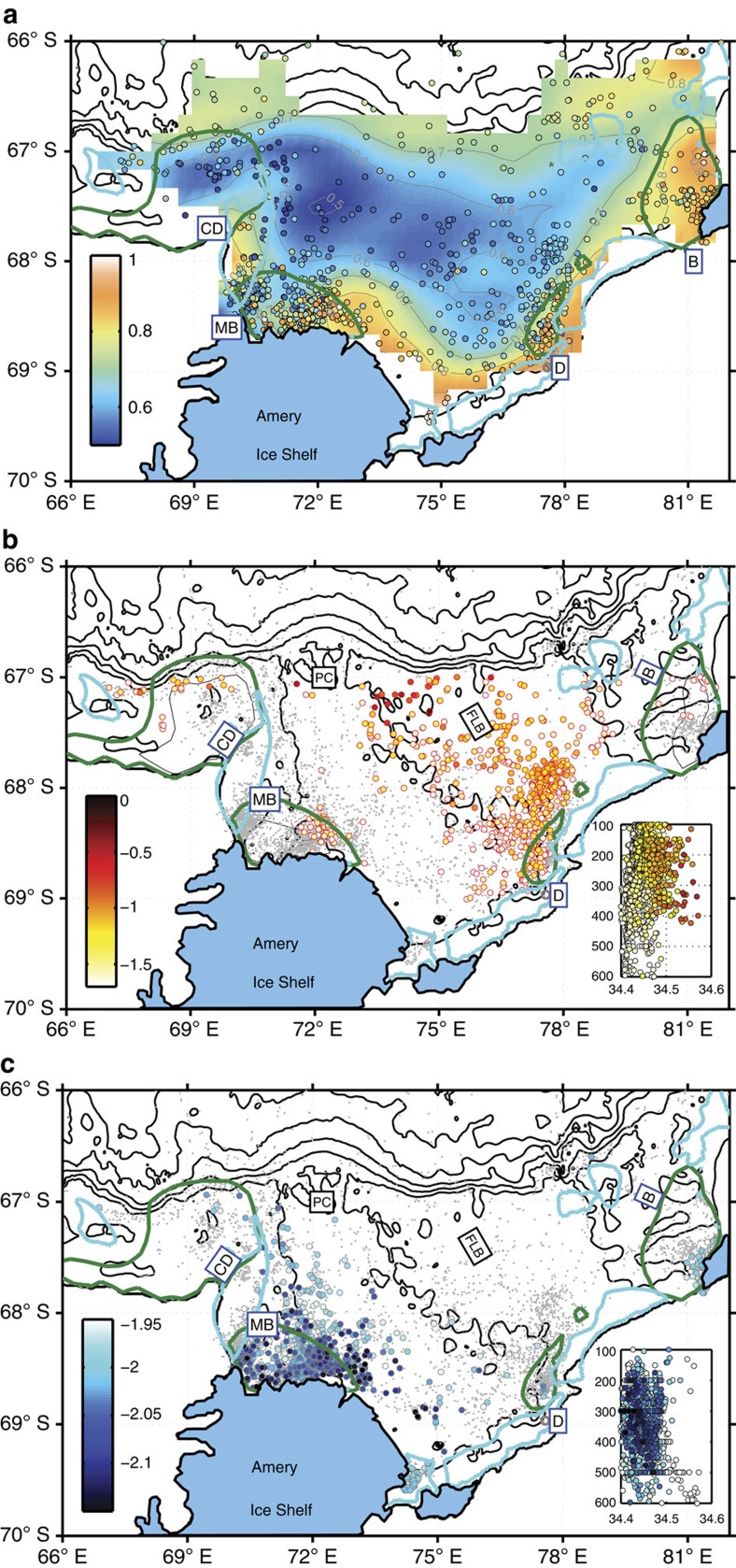
Circulation and water masses. (**a**) Geopotential anomaly (m^2^ s^−2^) at 50 m relative to 300 m, from elephant seal CTD profiles from April through May 2011 and 2012. Data shown as colour shaded circles and objectively mapped contours (within an error of 0.025 m^2^ s^−2^). Colour bar as shown. Coastal polynyas are labelled and shown using the satellite-derived sea ice production estimates (dark green contours show 5 ma^−1^) from ERA-Interim data (1992–2014)^14^,^15^: Cape Darnley (CD), MacKenzie Bay (MB), Davis (D) and Barrier Bay (B). Mean fast ice contours^37^ are shown as thick light blue contours. (**b**) Modified circumpolar deep water (mCDW). Maximum potential temperature (*θ* in °C) associated with intrusions of mCDW (circles, 28.0<*γ*^n^<28.27 kg m^−3^, −1.7<*θ*_max_<0 °C) into Prydz Bay from April through May. Profiles without an mCDW signal are shown as grey points. (**c**) Distribution of ice shelf water (ISW). Minimum potential temperature (*θ*_min_ °C, shaded in colour) associated with strong ISW signal (*θ*_min_<−1.95). Profiles without an ISW signal are shown as grey points. Insets in **b**,**c** show the vertical salinity profiles of mCDW and ISW, respectively.

It is somewhat counter-intuitive that the strongest DSW signal in Prydz Bay is in MacKenzie Bay, a region dominated by fresh ISW from the Amery Ice Shelf (Fig. 2c). However, as introduced in the previous section, a closer examination of the seal profiles relative to the ISW signal shows a strong gradient in water mass properties in the western corner of MacKenzie Bay (Supplementary Fig. 3). There appears to be an exclusion zone that the seals do not forage within, across which ISW is found to the east and the most saline DSW to the northwest (see May to June in Supplementary Fig. 3b). We speculate that this exclusion zone is a fast moving current of ISW from beneath the Amery Ice Shelf that produces the cooler, fresher DSW values in the eastern region of MacKenzie Bay. This current and associated front could explain the increase in DSW salinity to the west, relative to the freshening impact of ISW on the water column observed at PBM7 (Supplementary Fig. 4).

**Figure 3:**
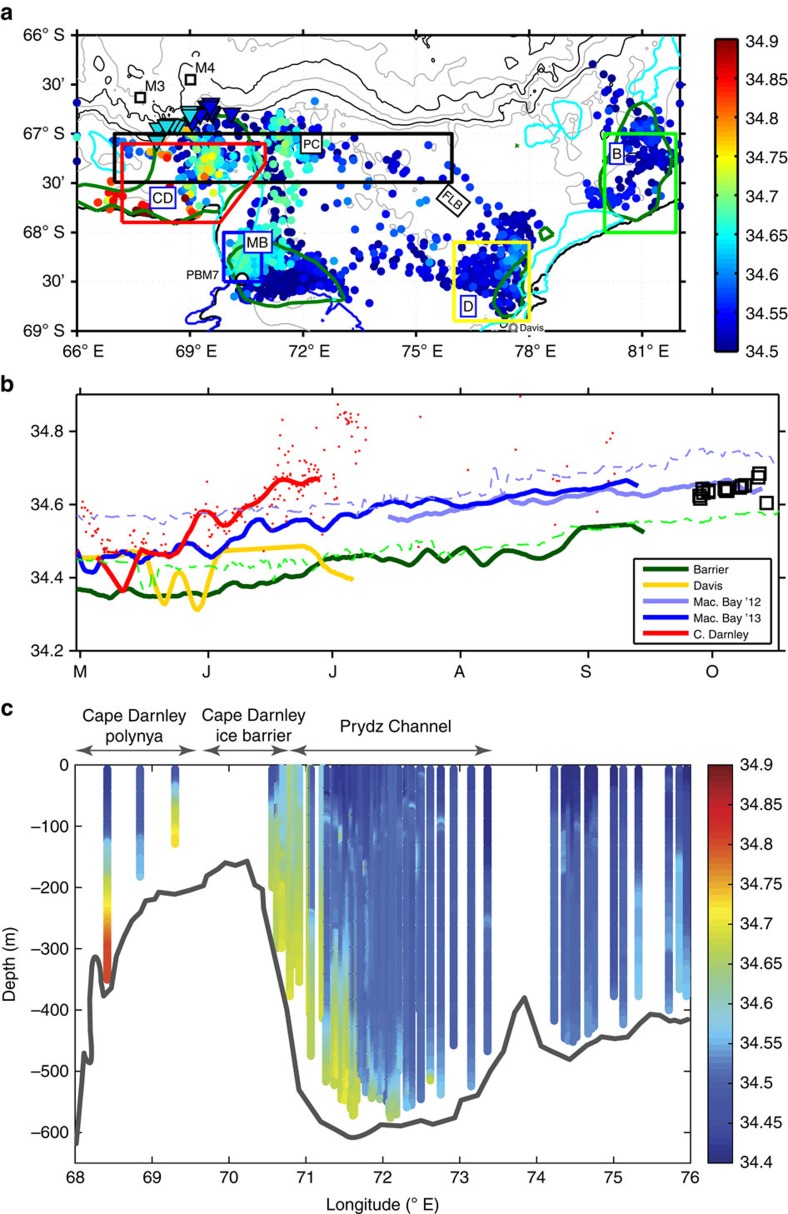
Dense shelf water around Prydz Bay. (**a**) Spatial distribution of bottom-of-dive salinity (coloured circles) corresponding to dense shelf water (DSW, *γ*^n^>28.27 kg m^−3^, *θ*<−1.8, salinity>34.4) from all seal data post-April 2011 to 2012. Cyan and dark green contours show fast ice and polynya regions, as in Fig. 1. Instrumented mooring locations M3, M4 (ref. 10) (black squares) and PBM7 (black circle) are shown. Inverted triangles show the locations of bottom modified Shelf Water (mSW) values from seal data post-September on the continental slope north of Cape Darnley (depth>800 m, *θ*<−0.8), split into saline (>34.6) values west of 69° E (cyan) and fresher (<34.6) values east of 69° E (blue). Coloured boxes with dashed lines show regional polynyas areas (light green, yellow, blue and red) used for subsequent time series analysis in **b**. Zonal black dashed box shows data region used for vertical section of export in **c**. (**b**) Time series of salinity at 300 m from seal occupations in key polynya areas (Barrier—dark green, Davis—yellow, MacKenzie—blue (2012) and light blue (2013) and Cape Darnley—red) demonstrating the regional variability in the formation of DSW. Time series presented as the 8-day running mean of available data around the 300 m layer, gridded at a half-day resolution. Additional time series are presented from oceanographic moorings in the western corner of the Amery Ice Shelf (PBM7—light green dashed line) and from the Adélie Sill of the Adélie Land region^8^ (light blue dashed line). Additional bottom-of-dive DSW data (from **a**) is shown for the Cape Darnley region (red dots) and along the western flank of the Prydz Channel (black squares). (**c**) Vertical section of salinity across the shelf break from 68 to 76° E in October highlighting DSW export from Prydz Bay east of 70° E. The primary outflow from Prydz Bay is in the bottom layer on the western flank of Prydz Channel, with some DSW on the western flank near the Cape Darnley ice barrier at 70.5° E. The most saline DSW, from Cape Darnley, is found west of 70° E.

The final part in this saga is the export of DSW from Prydz Bay. We trace the DSW signature from the western region of MacKenzie Bay out through Prydz Channel using a vertical section of salinity constructed from available profiles after 1 September along the shelf break from 68 to 76° E (Fig. 3c). The high-salinity signal (approaching 34.8) of DSW from Cape Darnley is detected in a profile near 68° E. But most importantly it clearly shows the first observational evidence of DSW export from Prydz Bay, in a 50–100 m thick bottom layer with salinities >34.65 between 70.5 and 72° E (Fig. 3c). This is sub-sampled from the annual distribution of DSW shown in bottom-of-dive salinities values in Fig. 3a. The DSW is following the western flank of the trough/sill, which rises to meet the eastern boundary of the Cape Darnley ice barrier (CDIB). We speculate that a component of this outflowing DSW could be flowing westwards beneath the fast ice of the CDIB, and that earlier in the season this transport could act to precondition/boost the DSW formed in the Cape Darnley polynya region.

**Figure 4:**
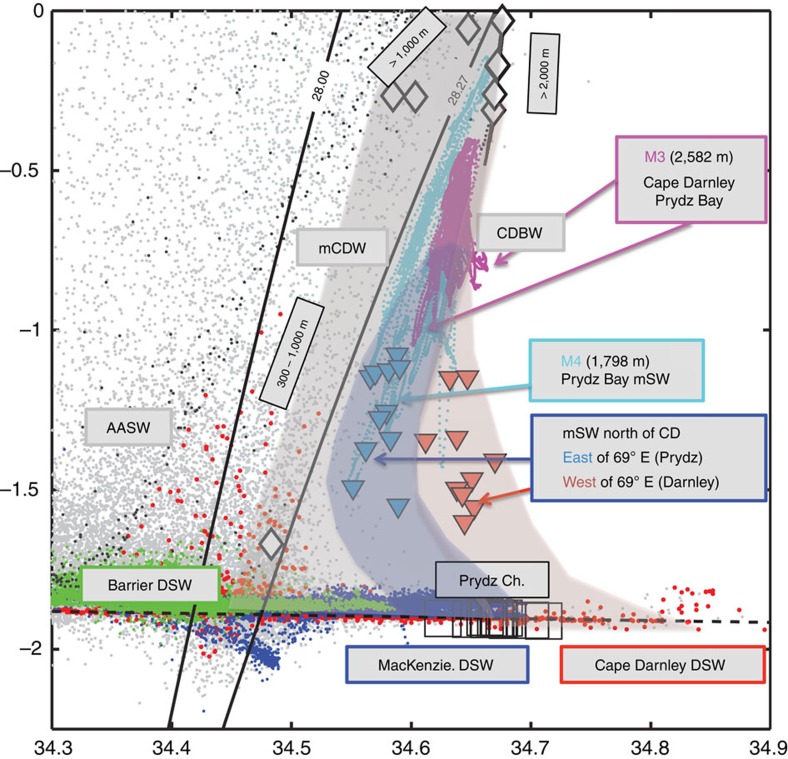
Dual contributions of DSW from Cape Darnley and Prydz Bay to CDBW. *θ*–S diagram showing regional water masses (AASW, Antarctic surface water; AABW, Antarctic bottom water; DSW, dense shelf water; mCDW, modified circumpolar deep water and mSW, modified shelf water). Proposed mixing pathways for DSW/mSW from Prydz Bay (shaded blue–grey) and Cape Darnley (red–grey), with the depth-varying properties of offshore mCDW (diamonds and light-grey shaded area) to produce CDBW. Standard neutral density contours broadly delineates the mCDW (28.00<*γ*^n^<28.27) from the AASW and the DSW/mSW/CDBW. All elephant seal data from 2011 to 2013 (Fig. 1) are shown as light-grey points. Dark-grey points are from BROKE-West, a shelf break/offshore summertime survey in 2006 from 30 to 80° E. DSW (Fig. 3) is found along the near-surface freezing point (black line) from Barrier Bay (green dots), MacKenzie Bay (blue dots) and Cape Darnley (red dots) polynyas. Bottom properties in Prydz Channel (from Fig. 3b), north of MacKenzie Bay, are shown as open black squares. White diamonds indicate mean properties from BROKE-West in the 100 m thick bottom layer of mCDW on the shelf break and continental slope at 70° E. Inverted triangles show bottom mSW values from elephant seals on the continental slope north of Cape Darnley^10^ (Fig. 3a, depth >800 m, *θ*<−0.8), split into saline (>34.6) values west of 69° E (light red) and fresher (<34.6) values east of 69° E (light blue). Data from M3 (magenta dots, 2,582 m) and M4 (cyan dots, 1,798 m) mooring data^10^, as indicated on Fig. 1.

With this first direct evidence of DSW export from Prydz Bay we can now present a complete description of the mixing pathways of dual contributions of DSW from Cape Darnley and Prydz Bay to CDBW in Fig. 4. DSW formation is shown along the near-surface freezing line, with the higher-/lower-salinity DSW in Cape Darnley (red)/Prydz Bay (MacKenzie Bay—blue; Barrier Bay—green), respectively, together with the DSW values exported through Prydz Channel (Fig. 4, black squares). Data from the near-bottom layer from offshore moorings at M3 (2,582 m) and M4 (1,798 m) are shown, overlain with modified shelf water (mSW) values from seal data on the continental slope south of these moorings^10^, as previously indicated with blue/light blue triangles in Fig. 3a. The mSW data, split into fresh (light blue)/saline (red) regions east/west of 69° E, line up with the mooring data at M4 and M3. This confirms the two curved mixing pathways of each independent DSW source from Cape Darnley and Prydz Bay (Fig. 4, light red and blue shaded areas, respectively), with the depth-varying properties of mCDW down the continental slope (grey-shaded area), to produce CDBW.

## Discussion

**Figure 5:**
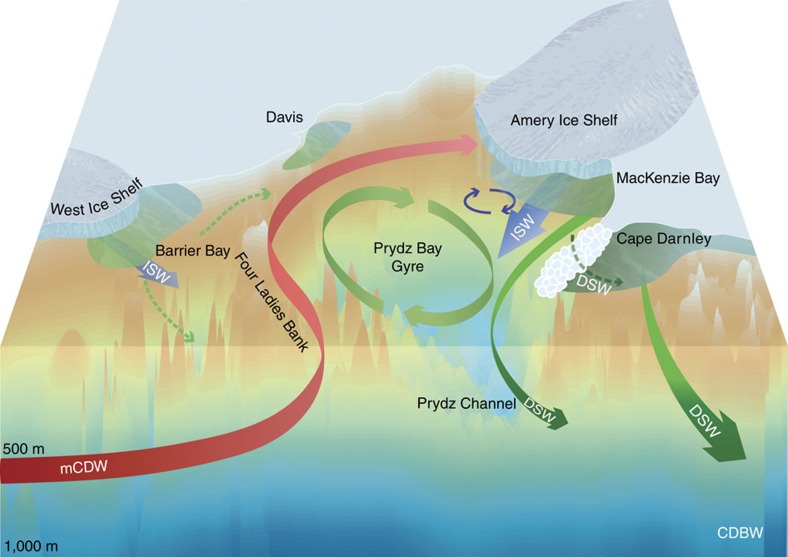
The evolution of Prydz Bay's dense shelf water contribution to Cape Darnley bottom water. From left to right (east to west), the major source water mass into Prydz Bay is the intrusion of mCDW over Four Ladies Bank and towards Davis Station and the eastern corner of the Amery Ice Shelf. While Barrier Bay is a significant polynya, its ability to form DSW is suppressed by ISW from the West Ice Shelf and it is bathymetrically isolated from the Prydz Bay Gyre. The strongest outflow of ISW into the region comes from beneath the Amery Ice Shelf, but it recirculates within the Prydz Bay Gyre and back under the Amery Ice Shelf. In the northwestern part of the MacKenzie Bay polynya region, DSW relatively unaffected by the ISW from the Amery flows out along the western flank and out through Prydz Channel, where after it migrates west and downslope to ultimately join the very saline DSW from Cape Darnley and produce new Antarctic bottom water to the Weddell–Enderby Basin. An additional pathway for DSW westwards beneath the Cape Darnley ice barrier (dashed arrow) is also shown.

Historically it has been speculated that Prydz Bay was a likely source of AABW to the Weddell–Enderby Basin^27^,^28^, given its broad physical similarities to the Weddell and Ross source regions. However, determining its exact DSW source remained elusive for many decades. Recent observations of newly ventilated AABW flowing downslope offshore from Cape Darnley^10^ shifted attention westward from Prydz Bay to Cape Darnley, after the CDBW was directly linked to very saline DSW (>34.8) in the Cape Darnley polynya. The influence of DSW from Prydz Bay was detected in a lower-salinity signature in offshore downslope flows of mSW north of Cape Darnley^10^. However, with limited observations from within Prydz Bay in winter, its contribution to CDBW was postulated to be weak. Now, with the expanded IMOS seal data sets from 2012 to 2013, we have presented the first complete regional assessment of Prydz Bay DSW over the entire winter period, including all the key polynya regions and most importantly—the export region through Prydz Channel (Fig. 5). We found the DSW exported from Prydz Bay to have salinity ∼34.67 during 2012–2013, that is, sufficiently dense to form AABW in its own right. This reaffirms the regional importance of Prydz Bay in the formation of dense shelf water and increases our expectations of its contribution to Cape Darnley bottom water.

The DSW ultimately exported from Prydz Bay results from a complex evolution as the original mCDW source water mass is transformed around a production line of coastal polynyas and ice shelves driven by the PBG (Fig. 5). Individually, the Barrier Bay, Davis and MacKenzie polynyas are medium sized, but their combined sea ice production of ∼194 km^3^ is ∼30% greater than Cape Darnley (Supplementary Fig. 1c; Supplementary Table 1). However, the overall salinity increase from brine-rejection in the Barrier Bay, Davis and MacKenzie polynyas is hindered by the freshening impact of the basal melting of the Amery Ice Shelf, and to a lesser extent the West Ice Shelf. At the end of the production line, the northwest region of the MacKenzie Bay polynya has the final input, increasing DSW salinities to values >34.67, akin to those exported from the Adélie Land region^8^ (Fig. 3b, light dashed blue line). The majority of this DSW flow along the western flank of Prydz Bay out towards the shelf break and continental slope, with some recirculation by the PBG back towards Amery Ice Shelf.

There are two possible pathways for the DSW from Prydz Bay *en route* to joining CDBW. Primarily DSW can flow directly out of Prydz Channel, and then migrate north-westward as a gravity current under the influence of the westward Antarctic Slope Current, as shown in Cape Darnley modelling studies^29^. In addition, some DSW could migrate westwards beneath the CDIB, to flow directly into the CDP region before leaving the continental shelf. The latter implies a direct pre-conditioning of the CDP, providing a ‘bump' or ‘assist' to the very saline values ultimately achieved in this region (*S*>34.8). It seems likely that a combination of both processes is occurring, given the low-salinity signal detected in the overflows of mSW north of Cape Darnley^10^. Both the CDP and the region surrounding the CDIB have largely unknown bathymetry, which has contributed to the unsuccessful retrieval of Japanese moorings deployed in this area during IPY. However, further mooring deployments are planned to measure and quantify the transport, if any, from Prydz Bay to Cape Darnley beneath the CDIB.

Recently, a warming trend (with no salinity change) has been documented in AABW properties downstream of the Prydz Bay–Cape Darnley system, due to increased entrainment of CDW^30^. It is postulated that fresher DSW advected downstream from the Indian–Pacific sector of the Southern Ocean may be offsetting a salinity increase due to more CDW. However, fresher DSW outflows from Prydz Bay, not yet considered, could also offset the salinity signal. In addition, the Barrier Bay polynya has significantly increased in size in the last decade, due to iceberg movement and fast ice changes^15^, and accordingly, its capacity for DSW formation and likely contribution to the interannual variability in AABW properties downstream. These studies highlight the importance of clearly understanding the variability of all components of CDBW, including the processes within Prydz Bay addressed in this paper.

As observational data sets expand into previously unexplored regions of the East Antarctic coastline, provided largely by animal-borne sensors, we are finding that each newly discovered polynya-based DSW region varies dynamically from the previous ones. In the Mertz Polynya, the large storage volume of the Adélie Depression aids the build-up of DSW^8^. Recently, the Vincennes Bay polynya, with relatively modest ice production and DSW formation, has been shown to be contributing mSW to the top layer of the offshore AABW^31^. The Cape Darnley polynya emerged with even stronger DSW properties, the limitation of its relatively narrow shelf region apparently overcome by the sheer strength of the sea ice production in the CDP^10^. Our new observations illustrate and reinforce the importance of upstream pre-conditioning and dual-sources of DSW in AABW production and highlight the complexities around AABW production dynamics.

The dual system of DSW contributions detailed in this paper for Cape Darnley and Prydz Bay is similar to other key AABW production regions around Antarctica. In the Ross Sea, there is high-salinity DSW input from Terra Nova Bay polynyas, together with a less dense, fresher variety of DSW from the Ross Ice Shelf^32^. Before the calving of the Mertz Glacier in 2010, there existed high-/low-salinity DSW export pathways from the Adélie and Mertz Depressions, respectively^9^. It could be argued there are multiple sources of DSW for the production of AABW in the Weddell Sea, including CDBW itself. Overall, the common factor for these regions is the freshening input from local ocean/ice shelf interactions, which as highlighted by this paper, can markedly suppress DSW formation.

A modelling study tested the impact of Antarctic ice shelf basal melting on deep ocean properties and showed that glacial basal melting stabilized the water column in front of an ice shelf as well as downstream, reducing the volume of DSW and thus of AABW^33^. The impact of basal melting occurred mainly in the Weddell Sea and the Ross Sea where large cavities are connected to broad continental shelves, however, the basal melt applied to the Amery Ice Shelf (17.65 Gta^−1^)^33^ was about half of the most recent estimate (39±21 Gta^−1^)^34^ and the low spatial resolution did not allow for a realistic representation of processes near the Amery Ice Shelf (for example, Prydz Bay polynyas). From our analyses, a slowing down of CDBW production as a result of increasing ice shelf melting seems very likely in the context of global climate warming.

To conclude, we provide novel evidence of dense shelf water export from Prydz Bay, demonstrating that it makes an important secondary contribution to Cape Darnley bottom water. The salinity of DSW leaving the MacKenzie Bay polynya is greater than 34.67, in the range of DSW exported from the Mertz Glacier region (∼142° E) in the production of Adélie Land bottom water. Cape Darnley DSW is the highest in salinity (34.8) and is clearly the primary source of CDBW, nonetheless, Prydz Bay makes a vital contribution to it, through the direct export of DSW through Prydz Channel and the potential pre-conditioning of the CDP beneath the Cape Darnley ice barrier. This study highlights the complex nature of DSW formation around Antarctica, in particular the importance of upstream regions on local water mass transformations and the likely impacts of enhanced ocean/ice shelf interactions. In the broader context of Antarctica's role in global climate, we find that despite strong sea ice production from three polynyas in the region, freshening from ocean/ice shelf interactions limits the overall formation of DSW in Prydz Bay.

There has been a lot of attention recently on the decadal-scale impact of icescape changes to AABW, resulting from major ice front calving events in polynyas regions, such as along Adélie Land after the calving of the Mertz Glacier^35^,^36^. This study suggests the more ubiquitous process of enhanced ocean/ice shelf interaction could be a far greater long-term threat to AABW production. Given the growing number of reports of accelerating and irreversible mass loss from Antarctica's major ice sheets linked to increased oceanic heat input, it is likely that Antarctica's AABW production is already compromised and will decrease further into the future.

## Methods

### Seal deployments

Instrumented elephant seals have been providing an invaluable additional source of oceanographic measurements to global databases, with particularly strong impact in data-poor regions such as around Antarctica^16^,^38^,^39^,^40^. These data fill important gaps in regions and seasons that are near impossible to observe by traditional ship-based methods, especially in winter. Here we use seal data from deployments of tags from Davis Station (2011 and 2012) and Kerguelen Island (2013) funded through the Integrated Marine Observing System (IMOS), focusing on the data returned from Prydz Bay and surrounds^41^. The 2012 and 2013 data provide expanded seasonal and spatial coverage, respectively, relative to the 2011 data previously reported^10^. These elephant seal data are publicly available from the MEOP consortium website (www.meop.net)^41^. Considerable effort has been undertaken to control the quality of the seal data given the obvious limitations relative to the WOCE-standard for ship-based measurements. Following ref. 41, we expect errors in the order of 0.03 for salinity and 0.02 °C for potential temperature. The time series of salinity evolution in the polynyas is constructed by sub-sampling for each region, interpolating the available 17-depth seal data profiles onto a 2 m grid and then taking the average across a 3-bin layer centered at 300 m. The maximum depth of 300 m was chosen to optimize the number of profiles available for the construction of the time series and to ensure a consistent comparison around Prydz Bay, where the bottom-of-dive depth for each seal varied seasonally and spatially. As a result, our estimate of DSW salinity is conservative because some deeper and more saline values were excluded.

### Data availability

The marine mammal data were processed and made freely available by the International MEOP Consortium and the national programs that contribute to it (http://www.meop.net). All relevant data are available from the authors.

## 

## Additional information

**How to cite this article:** Williams, G. D. *et al.* The suppression of Antarctic bottom water formation by melting ice shelves in Prydz Bay. *Nat. Commun.* 7:12577 doi: 10.1038/ncomms12577 (2016).

## Supplementary Material

Supplementary Information Supplementary Figures 1-4 and Supplementary Table 1
