# Supplementary material for: The suppression of Antarctic bottom water formation by melting ice shelves in Prydz Bay
Source: Nat Commun. 2016 Aug 23;7:12577. doi: 10.1038/ncomms12577 (PMC4996980; doi:10.1038/ncomms12577)
Supplement: Supplementary Information — Supplementary Figures 1-4 and Supplementary Table 1 [file ncomms12577-s1.pdf]

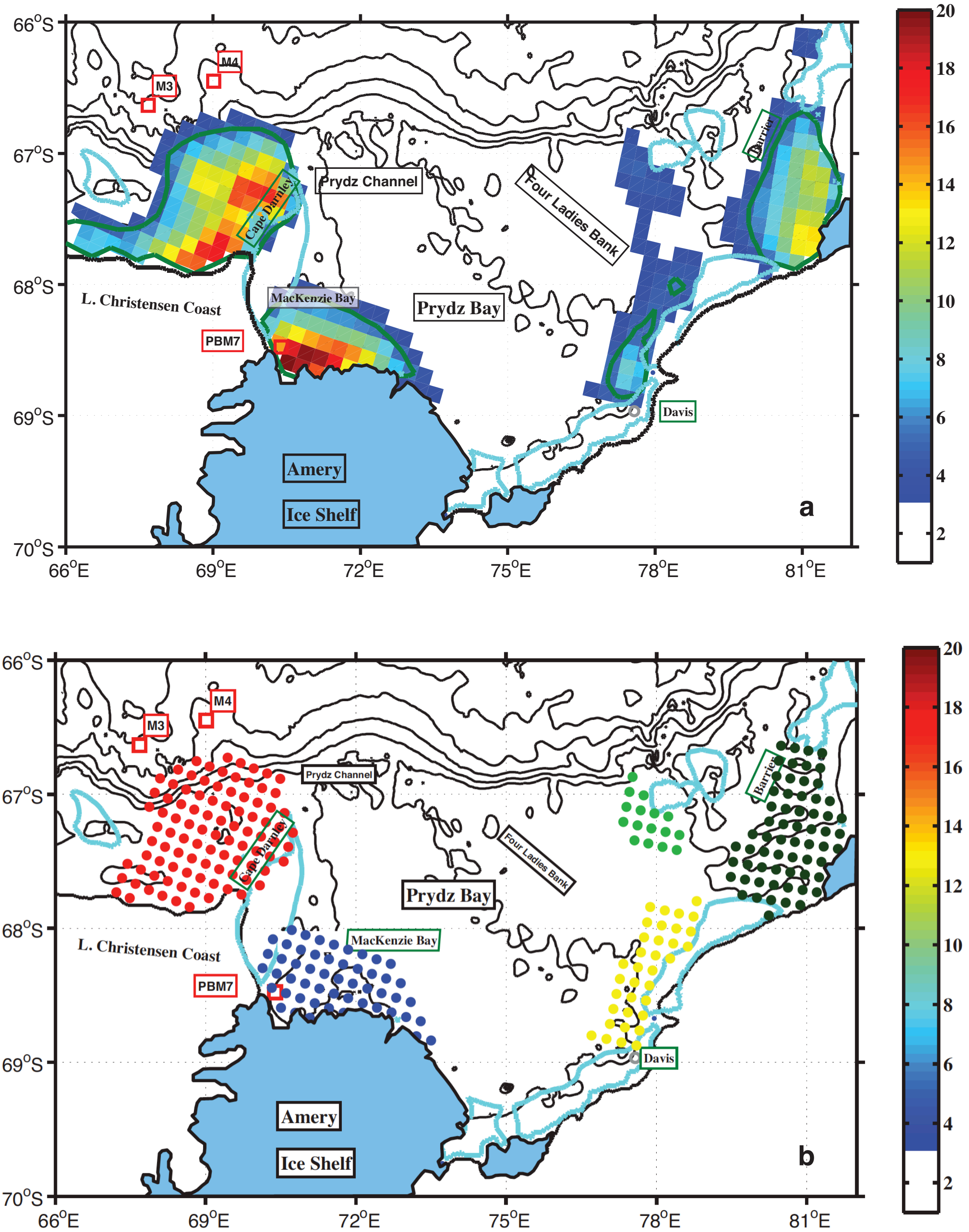

66°S
67°S
68°S
69°S
70°S
66°E
69°E
72°E
75°E
78°E
81°E
M3
M4
PBM7
Prydz Channel
Cape Darnley
Four Ladies Bank
Barrier
MacKenzie Bay
Prydz Bay
L. Christensen Coast
Davis
Amery
Ice Shelf
a
b
20
18
16
14
12
10
8
6
4
2

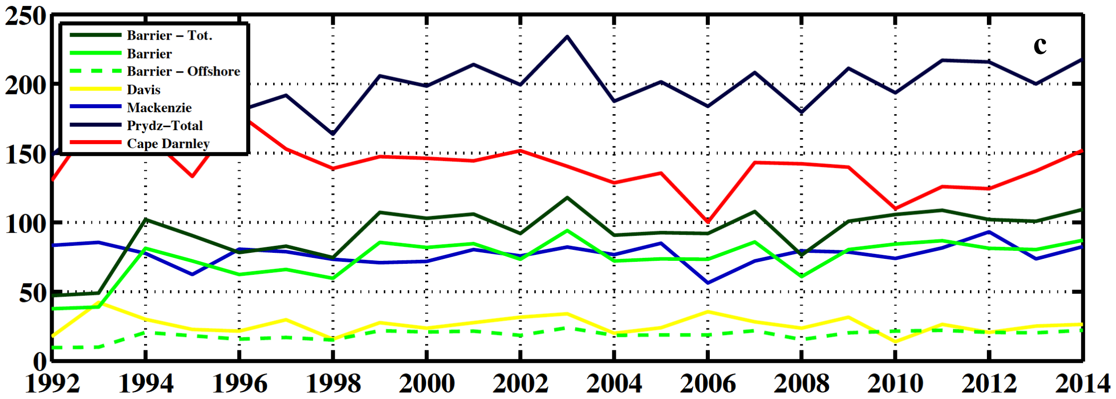

Figure 1. Regional distribution of satellite-derived sea-ice production. a Mean annual sea-ice production (SIP, m) from 1992—2014[15]. Features are as shown in Figure 1. **b** Regional polynyas masks based on 1992—2014 mean shown in **a**. Barrier Bay SIP split into coastal (dark green) and offshore (light green) components. **c** Annual satellite-derived SIP (km^3) averaged over each polynya region, as defined in **b**. Colours as indicated in legend. Total SIP for Prydz Bay (black) comprises of Barrier Bay – Total, Davis and Mackenzie Bay.

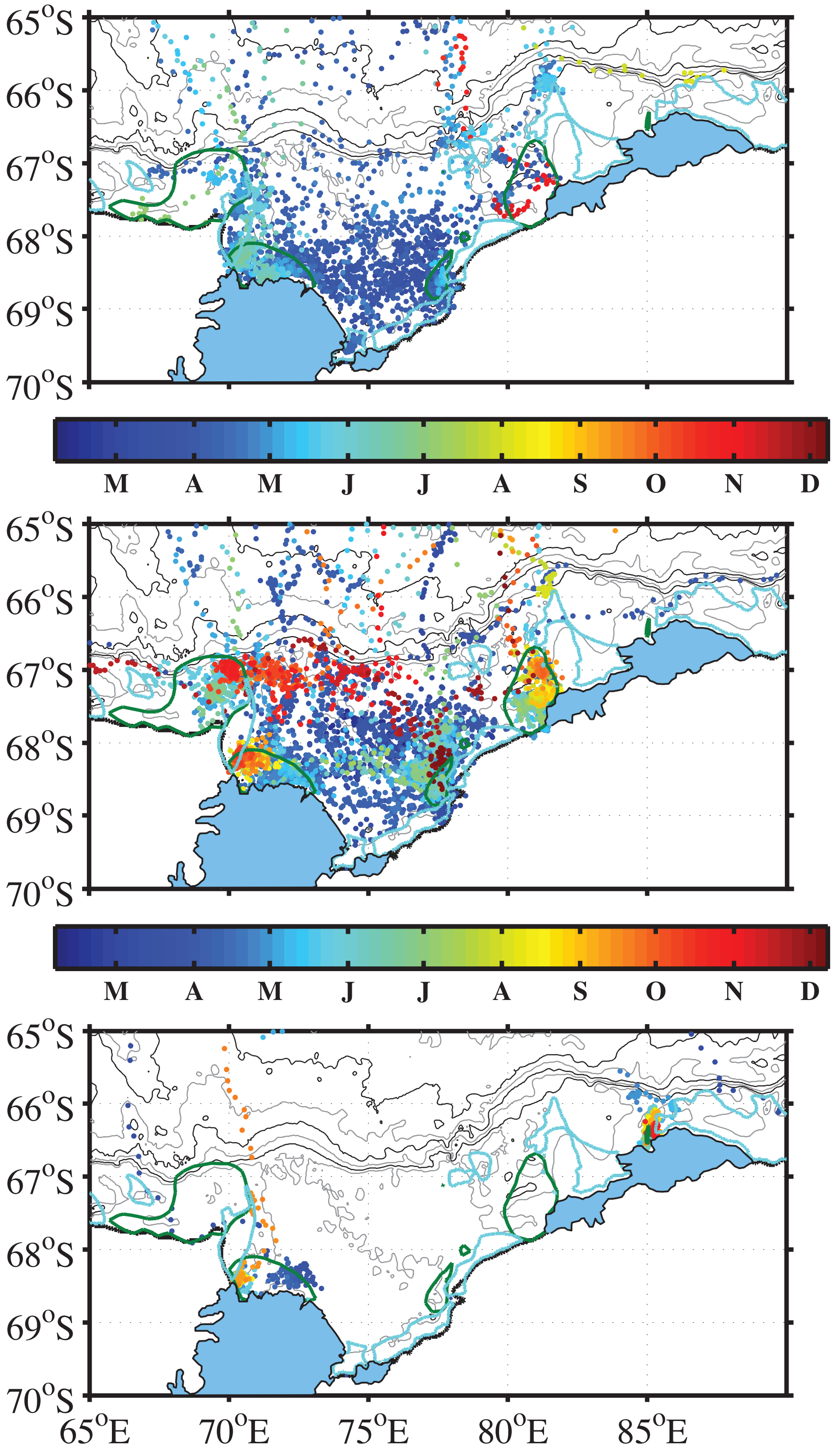

Figure 2. Seal data locations around Prydz Bay in 2011-2013. Seal locations shaded for day-of-year. Map features as in Figure 1. 2011—2012 deployments from

Davis Station (top and middle panel) and the 2013 deployments were from Isles Kerguelen (bottom panel).

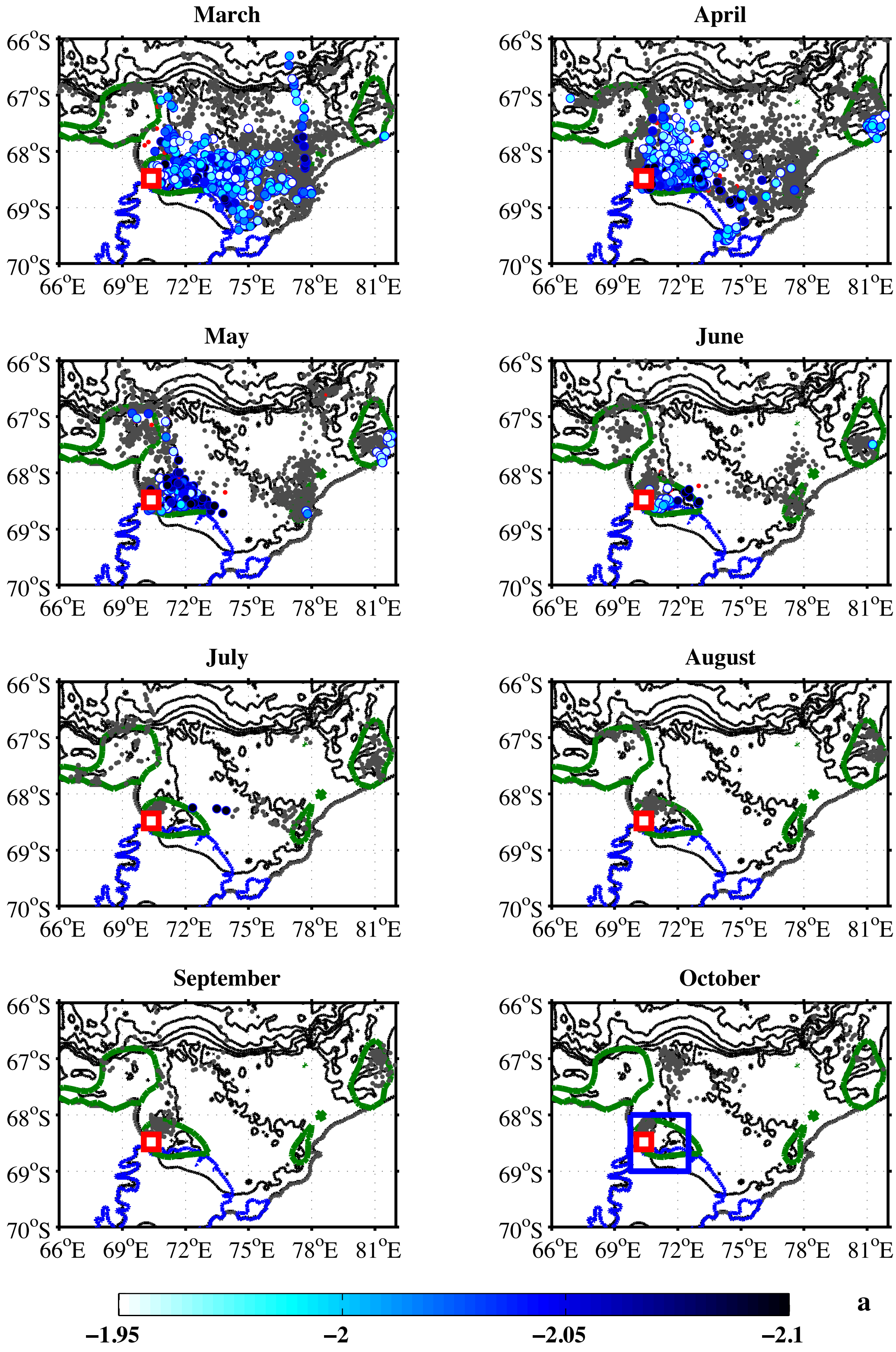

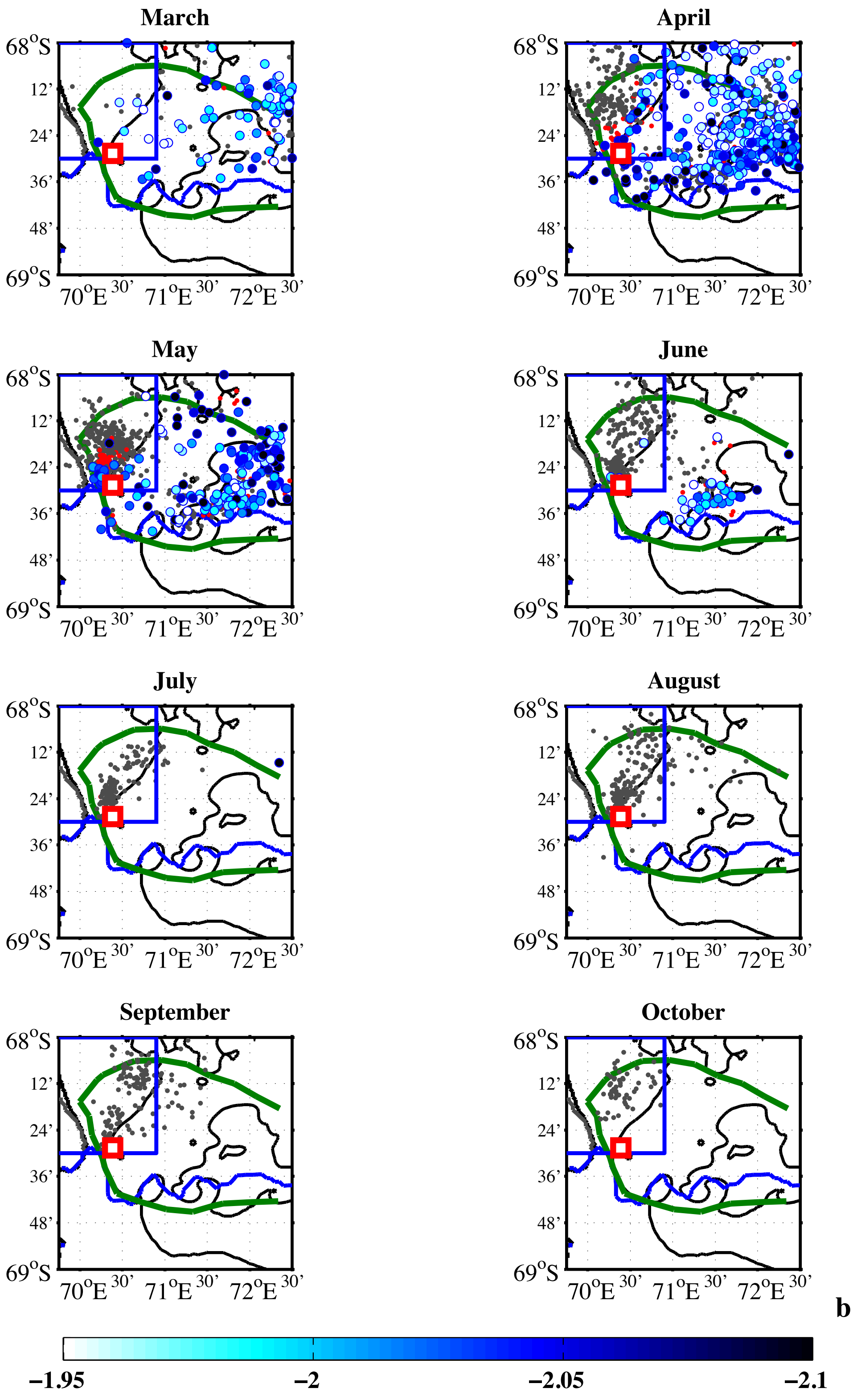

Figure 3. The spatial distribution of monthly ISW in Prydz Bay. a Profiles with a strong ISW signature (ISW layer thickness greater than 100m and minimum temperature <-1.95℃) are coloured as shown. Red dots represent profiles with a weak

ISW signature. Grey dots represent seal profiles that do not show the ISW signature. Red square is AMISOR mooring PMB7. Green contour is the Mackenzie Bay polynyas. Blue line is the Amery Ice Shelf front. **b.** As above, but focussed on the Mackenzie Bay polynya region in the western corner of the Amery Ice Shelf front. Blue box is outline of region used to construct DSW salinity time series for Mackenzie Bay in Figure 3b.

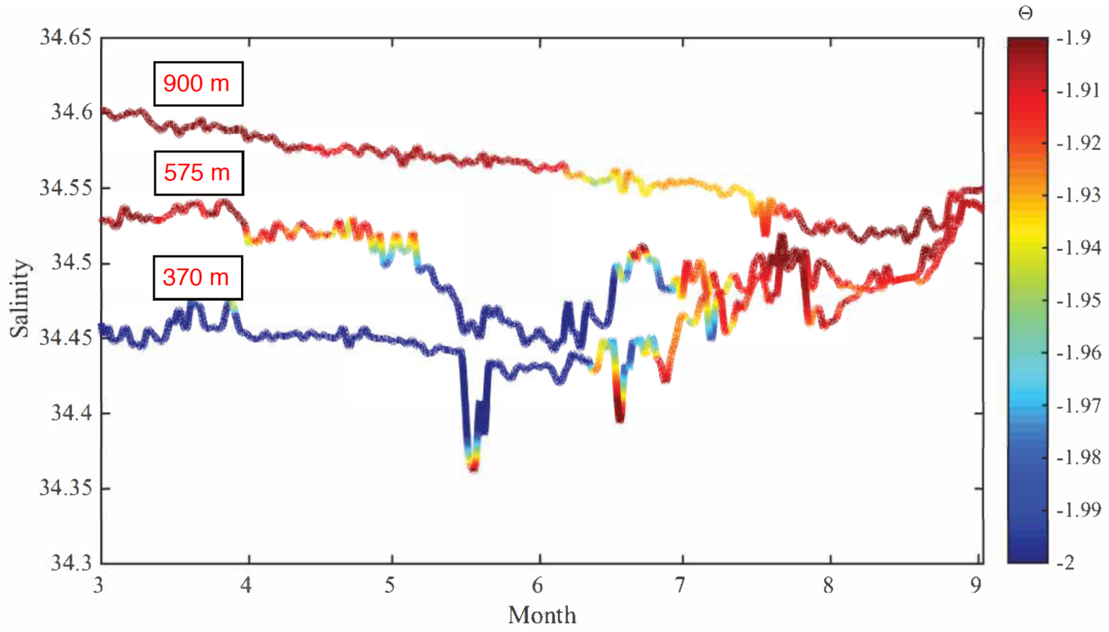

Figure 4. Time series of salinity at mooring PBM7. The time series are colored for potential temperature**.** Data shown at 370, 575 and 900m depths during 2001.

Table 1. Regional polynya sea-ice production rates.

**Polynya Region**	**Mean Annual SIP (1992-2014) km^3**	**St. Dev (1992-2014) km^3**
Barrier - Total	93	18
Barrier - Coastal	74	14
Barrier - Offshore	19	4
Davis	26	7
Mackenzie Bay	77	8
Cape Darnley	140	19
**Prydz Bay - Total**	194	20
